# Relationship between pediatric asthma and respiratory microbiota, intestinal microbiota: a narrative review

**DOI:** 10.3389/fmicb.2025.1550783

**Published:** 2025-05-09

**Authors:** Lian Liu, Wenqi Zhao, Han Zhang, Yunxiao Shang, Wanjie Huang, Qi Cheng

**Affiliations:** ^1^Department of Pediatrics, Shengjing Hospital of China Medical University, Shenyang, China; ^2^School of Clinical Medicine, Qilu Medical University, Zibo, China

**Keywords:** respiratory microbiota, intestinal microbiota, microbiome, pediatric asthma, gut-lung axis

## Abstract

Pediatric asthma is a common chronic airway inflammatory disease that begins in childhood and its impact persists throughout all age stages of patients. With the continuous progress of detection technologies, numerous studies have firmly demonstrated that gut microbiota and respiratory microbiota are closely related to the occurrence and development of asthma, and related research is increasing day by day. This article elaborates in detail on the characteristics, composition of normal gut microbiota and lung microbiota at different ages and in different sites, as well as the connection of the gut—lung axis. Subsequently, it deeply analyzes various factors influencing microbiota colonization, including host factor, delivery mode, maternal dietary and infant feeding patterns, environmental microbial exposure and pollutants, and the use of antibiotics in early life. These factors are highly likely to play a crucial role in the onset process and disease progression of asthma. Research shows that obvious changes have occurred in the respiratory and gut microbiota of asthma patients, and these microbiomes exhibit different characteristics according to the phenotypes and endotypes of asthma. Finally, the article summarizes the microbiota—related treatment approaches for asthma carried out in recent years, including the application of probiotics, nutritional interventions, and fecal microbiota transplantation. These treatment modalities are expected to become new directions for future asthma treatment and bring new hope for solving the problem of childhood asthma.

## 1 Introduction

Bronchial asthma, a prevalent chronic inflammatory airway disorder in the pediatric population (Asher et al., 2021), emerges as a significant contributor to public health challenges, manifesting through elevated school absenteeism rates, increased emergency department utilization, and higher hospitalization frequencies (Naja et al., 2018).

The microbiota constitutes a complex and dynamic community of bacteria, viruses, and fungi, archaea, and other microorganisms, encompassing both commensal and pathogenic species, that colonize the surfaces or internal environments of hosts (humans, animals, plants) or external ecosystems. The microbiome primarily refers to the genomic repertoire of the microbiota, though these terms are frequently used interchangeably (Berg et al., 2020). Characterized by individual specificity, dynamic variability, and niche heterogeneity (Cho and Blaser, 2012), the human microbiota rapidly establishes colonization in the gastrointestinal tract, respiratory system, and other anatomical microenvironments after birth, with its composition continuously shaped by host factors and environmental exposures. A growing consensus emphasizes that these microbial communities and their metabolites participate in critical host biological processes, including maintaining immune homeostasis and modulating metabolic pathways, which promote health under homeostatic conditions while driving disease pathogenesis during dysbiosis. Notably, microbiota dysbiosis, defined as an imbalance between beneficial and pathogenic species or aberrant abundance of specific commensals, demonstrates significant associations with pulmonary disorders, including asthma (Barcik et al., 2020), bronchopulmonary dysplasia (Pammi et al., 2019), cystic fibrosis (Françoise and Héry-Arnaud, 2020) and COVID-19 (Ancona et al., 2023).

In recent times, with the remarkable advancements in microbiome technology, our comprehension of the relationship between the human microbiota and health—disease states has been continuously enhanced (Escobar-Zepeda et al., 2015; Davidson and Epperson, 2018). The gut microbiota and respiratory microbiota, being crucial constituents of the human microbiota (Clavijo-Salomon and Trinchieri, 2025), play a pivotal role in maintaining the body’s immune equilibrium and fending off pathogen incursions. A growing body of evidence suggests that the dysbiosis of gut microbiota and respiratory microbiota might be a key potential factor precipitating childhood asthma (Thorsen et al., 2023; Henrick et al., 2021). Through the gut—lung axis (Budden et al., 2017), the metabolites and immunomodulatory signals of gut microbes can influence the immune status and inflammatory responses within the respiratory tract. Nevertheless, our current knowledge regarding the characteristics of microbiota at diverse age phases and in different anatomical sites, along with their precise mechanisms of action in the initiation and progression of asthma, still requires substantial improvement. In—depth investigations into the associations among gut microbiota, respiratory microbiota, and childhood asthma not only contribute to unravelling the pathogenesis of asthma but also hold the promise of opening up novel avenues for the prevention and treatment of childhood asthma. Consequently, a systematic review of the research progress in this domain bears significant theoretical implications and clinical application value.

## 2 Healthy gut and respiratory microbiota

The establishment of the normal microbiota is intricately linked to the development of various systems in children and the onset and progression of diseases, particularly respiratory allergic disorders. The gut microbiota and respiratory microbiota vary significantly with age and across different distribution sites. The gut—lung axis likely exerts a subtle and far—reaching influence on the body through mechanisms such as metabolism and immune circulation. Moreover, diverse microbiota, including bacteria, fungi, and viruses, interact with one another to jointly maintain a healthy micro—ecological environment.

### 2.1 Healthy gut microbiota

The human gut inhabits ~10^14^ bacteria (Bäckhed et al., 2005), as the body’s most densely colonized site (Sender et al., 2016). The dynamic development of the gut microbiota constitutes a central biological determinant in early-life programming, with its interaction mechanisms through the gut-lung axis with distal organs (e.g., pulmonary system) emerging as a critical frontier in host immunometabolic regulation. Following birth, the intestinal mucosal surface undergoes rapid microbial colonization (Leiby et al., 2018), establishing itself as a primary site for microbiota development. Neonatal gut flora, dominated by *Enterococcus*, *Escherichia*, *Streptococcus*, and *Rothia* (Actinobacteria), reflects an aerobic environment. By 2–4 months, colonization shifts to *Enterobacteriaceae*, *Bifidobacteriaceae*, and *Clostridiaceae*, signaling oxygen reduction and lactic acid metabolism. These taxa gradually decline until 18 months under the “healthy microbiota maturation” framework (Ximenez and Torres, 2017), paralleling increased microbial diversity observed in neonatal fecal analyses (Casaburi et al., 2021). This dynamic developmental progression drives the establishment of unique gut microbial profiles during infancy. During this period, the gut microbiota exhibits lower microbial diversity, structural instability, and marked inter-individual variability. By approximately three years of age, this developmental trajectory transitions toward stabilization, converging with adult-like microbial composition while establishing a unique gut ecosystem characterized by host-specific features (Koenig et al., 2011; Solís et al., 2010). In humans, fecal microbiota maturation occurs predominantly within the first several years of life, marked by the dominance of *Firmicutes* and *Bacteroidetes* phyla typically observed by 3 years of age (Teo et al., 2018; Yatsunenko et al., 2012). Emerging evidence suggests early microbial interactions may begin prenatally: placental bacteria in some preterm infants share origins with maternal oral microbiota (Ye et al., 2020), implying fetal-microbe crosstalk during embryogenesis (Casaburi et al., 2021).

Although existing research has predominantly focused on bacterial components of the gut microbiome, it is crucial to recognize that the viral component also constitutes a significant member of this microbial community. During the initial months of life, bacteriophages, akin to bacteria, exhibit host-specific distribution within the gut and play a pivotal role in regulating bacterial growth through mechanisms such as lysis and lysogeny (Shah et al., 2023). In 2008, a groundbreaking study by Breitbart et al. (2008) on the infant gut virome first revealed that the intestinal viral community of neonates at one-week postpartum displays remarkably low biodiversity, with bacteriophages dominating the composition. This specific distribution pattern may regulate the population structure and quantitative dynamics of symbiotic microbiota through niche competition. Recent research, leveraging large-scale viral metagenomic analyses of fecal samples from 1-year-old infants, identified approximately 10,000 viral genomes spanning 248 viral family-level clades (VFCs). Notably, over half of these VFCs represent previously undocumented taxonomic units, predominantly classified under the Caudoviricetes class (Shah et al., 2023). Nevertheless, significant gaps persist in understanding the assembly mechanisms of viral communities during critical developmental stages in early life. Combined with the limited coverage of viral genome databases, these challenges substantially hinder efforts to decode the organizational principles of the human virome.

### 2.2 The gut-lung axis

The gut-lung axis refers to a bidirectional communication network between the gut and the lungs (Budden et al., 2017), mediated by microbiota, metabolites, immune signals, and neuroendocrine pathways. The gut and lung microbiota collectively influence respiratory health and diseases [such as asthma (Kim et al., 2021) and respiratory infections (Zhao et al., 2023) by regulating the host immune system, metabolic pathways, and inflammatory responses].

This intricate cross-talk is mechanistically underpinned by specific microbial-derived components and host signaling molecules that mediate bidirectional organ interactions. Imbalanced gut bacteria (e.g., low diversity or harmful overgrowth) disrupt lung immunity via blood or lymph, while lung microbes affect inflammation through immune cells like dendritic cells. Gut bacteria also boost regulatory T cells (Tregs) (Arpaia et al., 2013) and reduce harmful Th2/Th17 inflammation (Atarashi et al., 2015). Key metabolites (e.g., SCFAs) balance immune responses by targeting receptors or enzymes [e.g., HDAC (Wang N. et al., 2024)], linking gut health to lung diseases like asthma.

### 2.3 Healthy respiratory microbiota

Conventional microbiological dogma long maintained the sterility of pulmonary environments (lower respiratory tract), until paradigm-shifting molecular analyses circa 2010 (Hilty et al., 2010) fundamentally redefined our understanding of airway microbiota composition propelling respiratory microbiome research into scientific prominence. Building upon these revelations, this section critically examines the dynamic colonization mechanisms and signature microbial profiles within healthy respiratory ecosystems, including bacteria (bacteriome), virus (virome) and fungi (mycobiome).

#### 2.3.1 Bacteria (bacteriome)

Respiratory bacterial colonization exhibits spatiotemporal heterogeneity across anatomical niches and age strata. Oropharyngeal specimens (via oral rinse sampling) demonstrate microbial predominance of *Prevotella*, *Veillonella*, and *Streptococcus* genera (Dickson et al., 2017). In contrast to the taxonomically rich ecosystems of gut and oropharyngeal microbiota, the lower respiratory tract (pulmonary microbiome) maintains reduced microbial biomass while exhibiting marked ecological heterogeneity. Developmental analyses reveal neonatal pulmonary colonization initiates with *Staphylococcus* or *Corynebacterium* predominance, undergoing successional displacement by *Alloiococcus* or *Moraxella* genera during microbiota maturation (Teo et al., 2015). The stabilized pulmonary microbiome in healthy individuals is characterized by tripartite dominance of the following bacterial phyla: Firmicutes (genus *Streptococcus*), Proteobacteria (genus *Acinetobacter*), and Actinobacteria (genus *Corynebacterium*) (Bassis et al., 2015; Segal et al., 2013; Venkataraman et al., 2015), with core genera (*Streptococcus*, *Veillonella*, *Prevotella*) maintaining trans-anatomical equilibrium (Dickson et al., 2017). *Dolosigranulum pigrum* was more abundant in younger individuals while remaining present across all age groups. In contrast, the nasopharyngeal microbiota of adolescents and adults (≥15 years) was characterized by a consortium of less common taxa, including *Anaerococcus (octavius)*, *Corynebacterium*, *Finegoldia magna*, *Lawsonella clevelandensis*, and *Peptoniphilus* (Odendaal et al., 2024).

#### 2.3.2 Virus (virome)

The groundbreaking advances in NGS have enabled scientists to systematically unravel the complex ecology of the human lung virome. Studies reveal distinct characteristics of respiratory viral composition in healthy populations: viral diversity is markedly reduced (Willner et al., 2009), particularly in pediatric cohorts where *Anapoviridae* dominates, with minimal presence of human herpesviruses. In healthy lung tissues, this viral family persists as the core eukaryotic virome, occasionally coexisting with *herpesviruses*, *papillomaviruses*, and retroviruses (Willner et al., 2009). Intriguingly, latent viral phases may confer protective benefits to hosts—by persistently stimulating interferon-gamma (IFN-*γ*) secretion and activating macrophages, thereby establishing a foundational immune defense barrier (MacDuff et al., 2015; Sun et al., 2015). This mechanism is experimentally validated: mice latently infected with murine herpesvirus exhibit enhanced resistance to *Listeria monocytogenes* infections (Barton et al., 2007). Notably, emerging evidence identifies a rich phage community within respiratory surfaces, hypothesized to comprise 19 core species (Willner et al., 2009; Lim et al., 2013). These phages orchestrate microbial equilibrium through dual strategies—precisely eliminating competitor bacterial strains via prophage release while dynamically regulating proliferation balance among niche-sharing bacteria, revealing intricate cross-kingdom interactions. Future investigations must elucidate the mechanisms underlying viral interactions with other pulmonary microbes, which promise to unlock novel therapeutic avenues for respiratory disease prevention and treatment.

#### 2.3.3 Fungi (mycobiome)

Current mycological research primarily employs targeted sequencing approaches, including analysis of the internal transcribed spacer (ITS) region and 18S rRNA genes, complemented by shotgun metagenomic sequencing (Carney et al., 2020). Despite growing research interest in this field (Cui et al., 2013; Huffnagle and Noverr, 2013), pulmonary fungal studies face persistent technical challenges: extremely low biomass, limited taxonomic diversity, inefficient DNA extraction, 18S rRNA amplification bias, and inconsistent nomenclature standards, collectively hampering accurate fungal database annotation (Iliev et al., 2012; Marsland and Gollwitzer, 2014; Carmody et al., 2013). Compared to the well-characterized bacteriome, the functional significance of fungi in pulmonary ecosystems remains underexplored.

Emerging evidence reveals distinct diversity patterns in healthy pulmonary mycobiomes that significantly differ from pathological states (Fodor et al., 2012; Lim et al., 2014; Delhaes et al., 2012). Core fungal communities are dominated by *Ascomycetes* (phylum level) and *Streptomyces* (genus level), followed by *Candida*, *Saccharomyces*, *Penicillium*, *Dictyostelium*, and *Fusarium* (Huang et al., 2020; Sharma et al., 2019), with *Candida* demonstrating the highest relative abundance (Delhaes et al., 2012; Charlson et al., 2012; Ghannoum et al., 2010). Additional commensal species include *Aspergillus*, Davidiellaceae (family level), and *Eurotium* (Charlson et al., 2012). Notably, cross-kingdom interactions between fungi and bacteria exhibit regulatory mechanisms: *Candida* shows positive correlation with *Lactobacillus* but negative association with *Helicobacter pylori*. Experimental evidence confirms that *Lactobacillus* suppresses the epithelial adhesion capacity of both *H. pylori* and *Candida albicans*, thereby modulating their colonization dynamics (Dohlman et al., 2022). Previous studies have systematically delineated the colonization dynamics and characteristic microbiota composition of the gut and respiratory tract during early life, demonstrating the pivotal role of these symbiotic microorganisms in maintaining homeostasis for proper immune system development. Importantly, emerging evidence suggests that disruption of this delicate microbial equilibrium by various factors—including prenatal, perinatal, and postnatal influences—may compromise immune regulatory pathways, thereby substantially elevating the susceptibility to childhood asthma.

## 3 The factors that trigger changes in the microflora and trace asthma origin

The colonization process and change in species composition of respiratory and gut microbiome in early life are significantly associated with asthma susceptibility. This dynamic process is jointly regulated by the host’s age and multiple factors spanning prenatal to postnatal stages, including biological elements such as delivery mode, maternal dietary and infant feeding patterns; ecological components like environmental microbial exposure and pollutants; as well as critical regulatory factors including early life antibiotic use. These multifaceted influences collectively shape microbial colonization and immune development, ultimately affecting asthma risk.

### 3.1 Host

Recent studies have revealed significant variations in the composition of respiratory microbiota based on the host’s age (gestational age). Comparative studies have demonstrated gestational age-dependent variations in microbial community composition, revealing developmental-stage-specific differences (Bargheet et al., 2023). Extremely preterm infants exhibit delayed gut microbiota maturation with higher abundances of potentially pathogenic bacteria like *Escherichia coli* and *Staphylococcus epidermidis* compared to full-term infants, who are dominated by beneficial *Bifidobacterium* and *Bacteroides* species. Very preterm infants show intermediate microbiota profiles, characterized by reduced diversity and delayed colonization of symbiotic bacteria, influenced by gestational age. The nasopharynx of younger individuals is dominated by *Dolosigranulum pigrum* (*D. pigrum*), a species universally present across all age groups but exhibiting significantly higher abundance in pre-adolescent populations.

### 3.2 Delivery mode

The mode of delivery significantly influences the early colonization and developmental trajectory of both respiratory and gut microbiota in infants. Systematic reviews have demonstrated that vaginally delivered newborns exhibit significantly higher gut abundance of *Actinobacteria*, *Bacteroides*, and *Bifidobacterium* compared to cesarean-delivered counterparts (Rutayisire et al., 2016). Recent studies further reveal distinct gut microbial profiles at one week of age: cesarean-born infants predominantly harbor *Citrobacter freundii*, *Clostridium* spp., and *Staphylococcus aureus*, whereas vaginal delivery promotes preferential colonization by *Escherichia coli* (Stokholm et al., 2020).

The mode of delivery also influences the types of respiratory microbiota. In the respiratory tract, vaginally delivered neonates display early colonization with *Corynebacterium* and *Dolosigranulum pigrum*. The sustained presence of these commensal bacteria is associated with enhanced microbial maturity and a marked reduction in long-term asthma risk (Chen et al., 2023). Longitudinal studies further show that vaginally born infants maintain higher abundance of *Bacteroides* by one year of age, with its deficiency being strongly correlated with asthma susceptibility and delayed microbial development (Chen et al., 2023). Conversely, cesarean-delivered infants exhibit characteristic respiratory microbiota delays: early enrichment of *Gemella* and *Streptococcus*, followed by aberrant proliferation of oral bacterial genera such as *Neisseria* and *Prevotella* (Bosch et al., 2017). This dysbiotic profile, coupled with reduced colonization of health-associated commensals (e.g., *Corynebacterium*), may increase susceptibility to respiratory diseases through immune dysregulation (Bosch et al., 2016). Although the impact of delivery mode on respiratory microbiota is less pronounced than on gut microbiota (Chu et al., 2017; Biesbroek et al., 2014), their interplay remains clinically significant. For instance, early asymptomatic colonization of the respiratory tract with *Streptococcus* during infancy has been identified as a strong predictor of asthma development, likely mediated by disrupted immune education through host-microbial interactions in early life (Teo et al., 2015).

### 3.3 Maternal dietary and infant feeding patterns

Maternal dietary patterns during pregnancy, combined with postnatal feeding strategies, collectively shape offspring microbiota development trajectories, thereby influencing the risk of asthma and allergic diseases. A nested cross-sectional study (the MAMI cohort) demonstrated that maternal dietary patterns during pregnancy significantly influence neonatal gut microbiota development. Specifically, high intake of saturated fatty acids (SFAs) and monounsaturated fatty acids (MUFAs) leads to an abnormal enrichment of *Firmicutes* in the infant gut. This microbial dysbiosis is negatively associated with high consumption of fiber, proteins from vegetable sources, and vitamins during pregnancy (Selma-Royo et al., 2021). Arpaia et al. further support the protective role of dietary fiber, which modulates the *Firmicutes*: *Bacteroidetes* ratio to influences allergic airway disease (Arpaia et al., 2013). These findings underscore the importance of optimizing maternal fatty acid intake and increasing fiber-rich foods during pregnancy to mitigate asthma risk.

Infant feeding patterns (postnatal nutrition) also plays a critical role in shaping microbial trajectories that influence disease susceptibility. Breastfeeding promotes the early colonization of beneficial respiratory commensals (e.g., *Corynebacterium* and *Dolosigranulum pigrum*) and enhances gut *Bifidobacterium* abundance (Chen et al., 2023; Bosch et al., 2017). In contrast, formula feeding is associated with enrichment of *Gemella*, *Streptococcus* in the infant respiratory microbiota and oral-type anaerobic bacteria such as *Prevotella* and *Neisseria* species, disrupting microbial stability (Bosch et al., 2017). Moreover, breast milk-derived hereditary microbes may further protect against pediatric asthma by enhancing intestinal immune tolerance (Fang et al., 2024).

### 3.4 Environmental microbial exposure and pollutants

Epidemiological evidence demonstrates that residential microbial exposure critically modulates pediatric asthma risk, with cohort studies revealing an inverse correlation between indoor microbial diversity and asthma incidence across distinct living environments such as traditional farms versus urban communities (Sun et al., 2023; Ege et al., 2011). This protective effect may stem from dynamic microbial colonization during immune system maturation, where gut microbiota play a pivotal role in immune education (Abt et al., 2012). However, environmental pollutants can disrupt this balance. For instance, PM2.5 alters gut microbiota composition by increasing *Bacteroidetes* and decreasing *Firmicutes*, a shift mechanistically linked to asthma development (Zhao et al., 2023). Animal studies further demonstrate that chronic PM2.5 exposure induces persistent gut-lung microbiota dysbiosis, contributing to late-onset asthma progression (Zhao et al., 2023). Similarly, prenatal and postnatal tobacco smoke exposure elevates *Enterobacteriaceae* abundance, exacerbating respiratory symptom risks in infancy (Vardavas et al., 2016). Notably, urban microbial diversity may counteract allergic risks. High-allergen environments combined with indoor dust microbial richness—particularly the presence of protective taxa such as *Prevotellaceae* and *Lachnospiraceae*—attenuate wheezing, suggesting a potential buffering effect against asthma-related outcomes (Lynch et al., 2014). Conversely, crowding conditions, including the presence of young siblings or daycare attendance, reduce respiratory microbiota stability and promote *Pasteurellaceae* (e.g., *Haemophilus*) dominance (Bosch et al., 2017). These findings collectively highlight the delicate balance between protective microbial exposures and pollutant-driven dysbiosis in shaping asthma pathogenesis.

### 3.5 Early life antibiotic use

Recent studies have revealed that early-life antibiotic exposure profoundly influences asthma development by altering microbiome composition. The widespread use of antibiotics during pregnancy and infancy not only promotes the emergence of multidrug-resistant pathogens (Alfaqawi et al., 2021), complicating asthma treatment, but also directly disrupts the dynamic balance of respiratory and gut microbiota in affected individuals. Clinical evidence indicates that antibiotics significantly deplete key commensal bacteria in infant airways, such as *Corynebacterium* and *Alloiococcus*, leading to reduced microbial community stability (Bosch et al., 2017). This microbial dysbiosis extends to the gut, where antibiotic intervention promotes abnormal yeast proliferation, thereby exacerbating pulmonary allergic responses (Noverr et al., 2004). Animal studies provide direct evidence for the impact of early-life antibiotic exposure on asthma pathogenesis. Neonatal mice exposed to azithromycin or amoxicillin exhibited marked reductions in the diversity of core gut microbial taxa, including *Lachnospiraceae* and *Muribaculaceae* (Borbet et al., 2022). Such microbial disturbances have long-term consequences—when these mice were later exposed to house dust mite allergens, they displayed elevated IgE and IL-13 levels (hallmark biomarkers of allergic asthma), alongside hyperactivation of Th2/Th17 immune pathways and significantly enhanced airway hyperreactivity. Notably, microbiota transplantation experiments demonstrated that offspring of germ-free mice colonized with gut microbiota from antibiotic-exposed mice developed hyperactive immune responses and asthma-like symptoms despite no direct antibiotic exposure, suggesting that early-life microbial alterations can program immune development through transgenerational transmission. Animal studies have shown that antibiotic use not only causes intestinal bacterial dysbiosis increasing asthma risk, but also induces intestinal fungal dysbiosis. Kim et al. found that combined antibiotic use leads to overgrowth of the commensal fungus Candida in the gut. Candida promotes M2 macrophage polarization in the lungs by elevating plasma prostaglandin E2 (PGE2) levels, thereby increasing airway inflammatory cell infiltration and exacerbating tissue pathological changes (Kim et al., 2014).

## 4 Microbial alterations in pediatric asthma

Pediatric asthma demonstrates distinct gut-respiratory microbiota compositional differences compared to healthy controls. In this section, we review airway and gut microbial alterations in pediatric asthma, and microbial changes associated with asthma phenotypes and endotypes.

### 4.1 Intestinal microbita in pediatric asthma

The gut’s dysbiosis may disrupt immune homeostasis, and specific microbial shifts correlate with asthma development. Multicenter cohort studies revealed elevated *Prevotella* (*P. bivia*, *P. disiens*, *P. oris*) and *Bacteroides fragilis* colonization across oral-gut ecosystems, coupled with reduced *Streptococcus thermophilus* levels (Yan et al., 2024). The dynamic changes in gut microbiota during early life are closely associated with the progression of asthma (Rautava and Walker, 2009; Simonyte Sjödin et al., 2016). The Copenhagen Prospective Studies on Asthma in Childhood (COPSAC) (Stokholm et al., 2018) demonstrated that early-life gut microbial alterations in 1-year-old infants born to asthmatic mothers, characterized by dysregulated relative abundances of *Veillonella*, *Lachnospiraceae*, *Bifidobacterium*, and *Ruminococcus*, served as significant predictors of asthma development by age 5. The Canadian Healthy Infant Longitudinal Development (CHILD) Study (Arrieta et al., 2015) further identified the first 3 months of life as a critical window for gut microbiome-host interactions during which abnormal perturbations in gut microbiota structure exert profound long-term effects on airway health. Animal experiments using germ-free mice inoculated with stool from infants with atopic wheeze showed that supplementation with these depleted bacterial taxa reduced lung inflammation, elevated SCFA levels, and lowered proinflammatory cytokines in offspring (Cait et al., 2018; Trompette et al., 2014). SCFAs—produced through bacterial fermentation of dietary fiber —promote Treg differentiation (Roduit et al., 2019) via histone deacetylases (HDAC) inhibition, suppress M2 macrophage activation, and exert transgenerational protective effects (Calışkan et al., 2013; Wang et al., 2023) when supplemented during pregnancy (Arpaia et al., 2013; Trompette et al., 2014; Roduit et al., 2019; Calışkan et al., 2013; Huang et al., 2022). These findings collectively highlight the protective role of specific gut microbiota and their metabolites (e.g., SCFAs) in asthma pathogenesis.

Compared to the well-established gut microbiota-asthma research, studies on gut viruses and fungi are both fewer in number and more preliminary in nature. The virome comprises a small proportion of the overall gut microbiome. Recent study revealed associations between the infant gut virome composition and the risk of developing asthma (Leal Rodríguez et al., 2024). Bacteriophages, also known as phages, are viruses that infect and replicate within bacterial cells and are important determinants due to their ability to infect other bacteria, while they serve as mediators between pathogenic and nonpathogenic bacteria (Sweere et al., 2019). Specific temperate bacteriophage taxa, particularly 19 caudoviral families, were found to contribute to asthma risk. Children who later developed asthma exhibited lower relative abundances of these temperate phage families, which predominantly infect bacterial genera such as *Faecalibacterium* and *Ruminococcus*. Intriguingly, the virome-asthma association was independent of bacterial communities, with additive effects observed when combining virome and bacteriome signatures. The study also identified a potential interaction between the virome and the host immune system via the *TLR9* rs187084 genetic variant, suggesting phage DNA may directly modulate immune responses. This study represents one of the few published investigations focusing on the intestinal virome in pediatric asthma pathogenesis. Although this is an observational study, its findings provide evidence for regulating gut phages through phage therapy or perinatal intervention and restoring the balanced temperate virome required for immune maturation as a novel preventive strategy for asthma. Future research should explore the diversity of gut viruses and fungi, their microbial interactions, and the underlying mechanisms in asthma development.

### 4.2 Respiratory microbiota in pediatric asthma

Significant differences exist in respiratory microbial composition between healthy children and asthma patients. In asthmatic children exhibit nasal microbiota enriched with *Moraxella* species [e.g., *Moraxella catarrhalis* (Raita et al., 2021)], whose relative abundance positively correlates with increased asthma exacerbation frequency (Zhou et al., 2019; McCauley et al., 2019; Durack et al., 2018). Conversely, enrichment of *Corynebacterium* and *Dolosigranulum* is associated with improved asthma control, reduced BAL eosinophil percentages, and decreased levels of pro-inflammatory factors such as IL-17 and IL-121 (McCauley et al., 2019; Durack et al., 2018). This microbial dysbiosis interacts closely with host immune responses: rhinovirus (RV) infection combined with colonization by *M. catarrhalis* or *Streptococcus pneumoniae* synergistically exacerbates asthma symptoms (Kloepfer et al., 2014), while early-life (1-month-old) upper respiratory colonization with *S. pneumoniae*, *Haemophilus influenzae*, or *Moraxella* significantly elevates asthma risk by age 5 (Thorburn et al., 2015). This effect exhibits atopy dependency—children with early atopic sensitization are more prone to developing “persistent wheeze” (Teo et al., 2018). Longitudinal studies demonstrate that seasonal fluctuations in nasal microbiota linked to virus-induced asthma exacerbations are further associated with the RV-C endotype, characterized by Moraxella-dominant communities, which significantly elevates recurrent wheeze risk (Raita et al., 2021; McCauley et al., 2022). In the lower respiratory tract, protective associations are observed in healthy children, where bronchial *Actinomyces* and nasal *Corynebacterium* negatively correlate with pro-inflammatory gene expression (Chun et al., 2020).

The respiratory virome also plays a critical role in asthma pathogenesis. Extensive research has demonstrated a strong association between pediatric asthma and respiratory viruses, with respiratory syncytial virus (RSV) and RV identified as key pathogenic drivers of asthma exacerbations (Korten et al., 2016; Sigurs et al., 2000; Rosas-Salazar et al., 2023). A prospective cohort study of 1,946 healthy term infants in the United States revealed that children uninfected with respiratory syncytial virus (RSV) during infancy had a lower incidence of asthma at age 5 compared to the infected group, and preventing RSV infection could reduce approximately 15% of pediatric asthma cases (Rosas-Salazar et al., 2023). RV is the most frequently detected pathogen in wheezing children over 1 year old, and its early-life infections (particularly repeated detection within the first 3 postnatal weeks) increase pre-2-year wheezing risk by 16% (Takashima et al., 2023). Lower respiratory RV infections within the first 3 years of life elevate asthma risk by 40-fold before age 6, far exceeding the effects of RSV infection (Jackson et al., 2008; Kusel et al., 2007; Psarras et al., 2006). Mechanistically, RV promotes airway remodeling by inducing growth factors (e.g., lumican, collagen I/V) and prolongs bronchial hyperreactivity via non-Th2-IFN pathways, with impaired Th1/IL-10 responses exacerbating this pathology in atopic individuals (Psarras et al., 2006; Skevaki et al., 2012; Spector et al., 2023; Xepapadaki et al., 2005; Megremis et al., 2018; Georgountzou et al., 2021). Additionally, asthmatic children display upper respiratory virome dysbiosis dominated by eukaryotic viruses and low bacteriophage abundance, while diminished antiviral cytokine responses correlate with high RV loads (Megremis et al., 2023; Rovira Rubió et al., 2023).

In fungal communities, the upper respiratory tract of asthmatic children is dominated by *Malassezia globosa* and *Malassezia restricta*. High baseline *M. globosa* abundance delays asthma control loss, whereas its reduced abundance during exacerbation correlates with heightened severe attack risk. Transition from well-controlled to uncontrolled asthma involves synchronized increases in fungal and bacterial diversity, suggesting fungal-bacterial interactions in disease progression (Yuan et al., 2023).

These findings highlight the multi-layered role of respiratory microbiota in asthma pathogenesis: bacterial dysbiosis modulates local immunity, viral colonization drives direct epithelial damage and immune remodeling, and fungal-bacterial crosstalk potentially shapes asthma phenotypes. These microbial signatures offer promising biomarkers for early prediction and targeted interventions in asthma management.

### 4.3 Gut and respiratory microbiota in pediatric asthma phenotypes and endotypes

Asthma, as a heterogeneous disease, encompasses different endotypic and phenotypic classifications (Akar-Ghibril et al., 2020), with corresponding variations in its microbial ecosystem dynamics. Table 1 presents an example of current asthma phenotypes as they relate to inflammatory endotypes (type 2-high or type 2-low) and phenotypic characteristics. Based on immune characteristics, it can be categorized into T-helper lymphocytes 2 high (Th2-high) and non-T-helper lymphocytes 2 high (non-Th2 endotypes) (Agache et al., 2021; Liang et al., 2022; Svenningsen and Nair, 2017). Based on inflammatory cell infiltration in induced sputum, asthma can be classified into four distinct inflammatory phenotypes: eosinophilic asthma, neutrophilic asthma, mixed granulocytic asthma, and paucigranulocytic asthma (Svenningsen and Nair, 2017). Th2-high endotype (often referred as eosinophilic asthma), driven by IL-4, IL-5, and IL-13-mediated Th2 polarization (Castan et al., 2020), exhibits selective enrichment of pathogenic genera within *Firmicutes* and Proteobacteria—including *Haemophilus*, *Neisseria*, *Streptococcus*, and *Moraxella*—alongside significant depletion of *Lactobacillus* (Durack et al., 2017). These microbial alterations correlate with elevated blood and sputum eosinophil levels (Zhang et al., 2016; Diver et al., 2022), with exacerbations marked by *Haemophilus* and *Staphylococcus* expansion paralleling Th2 cytokine surges, while stable phases favor anti-inflammatory commensals like *Corynebacterium* and *Prevotella* (Diver et al., 2022). Further investigations demonstrate seasonal variations in nasal microbiota linked to viral-induced asthma exacerbations, particularly highlighting that the RV-C endotype—characterized by *Moraxella*-dominated microbial composition, heightened Th2 cytokine levels, and modified lipid metabolites—significantly elevates the likelihood of recurrent wheezing episodes (Raita et al., 2021; McCauley et al., 2022). Experimental studies using *Aspergillus fumigatus*-induced allergic asthma models reveal that intestinal colonization by *Candida albicans* amplifies eosinophil and mast cell populations in bronchoalveolar lavage fluid (BALF), stimulates secretion of IL-5, IL-13, and interferon-gamma, and consequently intensifies pulmonary allergic immune reactions (Noverr et al., 2004).

**Table 1:** Endotypes and phenotypes of asthma.

Endotype	Phenotypic characteristics	Phenotype※	Clinical characteristics
Type 2 (T2)-high asthma IL-4, IL-5, IL-14	Blood eosinophilia (≥150 cells·μL-1) (Kuruvilla et al., 2019)	Atopic	Well defined, early onset, steroid sensitive
	Elevated tissue eosinophilia	Late onset	±concomitant CRSwNP, steroid refractory
	Elevated serum IgE (surpassing the normal range of 1.5-114kU·L-1) (Zetterström and Johansson, 1981; Françoise and Héry-Arnaud, 2020)	AERD	Adult onset
	Elevated FeNO (>19.5 ppb) (Holguin et al., 2020)		
	Upper airway comorbidities, including AR and CRSsNP/CRSwNP		
	Other type 2 comorbidities, including EoE and AD		
	Responsive to corticosteroids		
Type 2 (T2)-low asthma	Low blood eosinophil counts (<150 cells·μL-1#)	Non-atopic	Adult onset-paucigranulocytic or neutrophilic
	Sputum neutrophilia (>40% of total cells) (Kuruvilla et al., 2019; Arron et al., 2014; Carr et al., 2018)	Smokers	Older adults
	Obesity associated	Obesity-related	Female sex
	Poor response to corticosteroids	elderly	> 50 to > 65 years at onset

IL: interleukin; Ig: immunoglobulin; FeNO: fraction of exhaled nitric oxide; AR: allergic rhinitis; CRSsNP: chronic rhinosinusitis without nasal polyps; CRSwNP: chronic rhinosinusitis with nasal polyps; EoE: eosinophilic oesophagitis; AD: atopic dermatitis. ^#^: value within the normal range does not exclude atopy (Françoise and Héry-Arnaud, 2020).^※^: The phenotypic classification in this table is based on the article by Kuruvilla et al. (2019).

Conversely, non-Th2 endotype, or non- eosinophilic asthma, encompassing neutrophilic asthma mediated through IL-6/IL-17-driven Th1/Th17 pathways and paucigranulocytic asthma in which neither eosinophils nor neutrophils are increased (Tliba and Panettieri, 2019; Hammad and Lambrecht, 2021). Neutrophilic asthma shows reduced microbial diversity with predominant *Haemophilus* and *Moraxella* enrichment in Proteobacteria, alongside dynamic *Haemophilus-Staphylococcus* fluctuations during exacerbations (Diver et al., 2022). A Proteobacteria-dominant microbial profile was associated with sputum neutrophilia, but also a longer duration of disease (Diver et al., 2022). The non-Th2 frequently displays corticosteroid resistance and severe clinical manifestations (Moore et al., 2014). Phenotypically, the enrichment of *Haemophilus* and *Neisseria* (phylum Proteobacteria) in the respiratory tract of children with asthma is associated with reduced post-bronchodilator FEV_1_/FVC ratio, mixed granulocytic asthma, and activation of the PD-L1/Th2 pathway (Kim et al., 2023). While nasal dominance of *Moraxella* and *Alloiococcus* coupled with bronchial *Actinomyces*-mediated anti-inflammatory regulation (Chun et al., 2020) further underscores microbial modulation of disease trajectories. Pediatric asthma highlights these interactions through heightened microbial-host crosstalk, where specific taxa influence inflammatory gene networks (Chun et al., 2020), collectively illustrating the respiratory microbiome’s multidimensional role in shaping asthma endotype-phenotype interrelationships.

**Figure 1:**
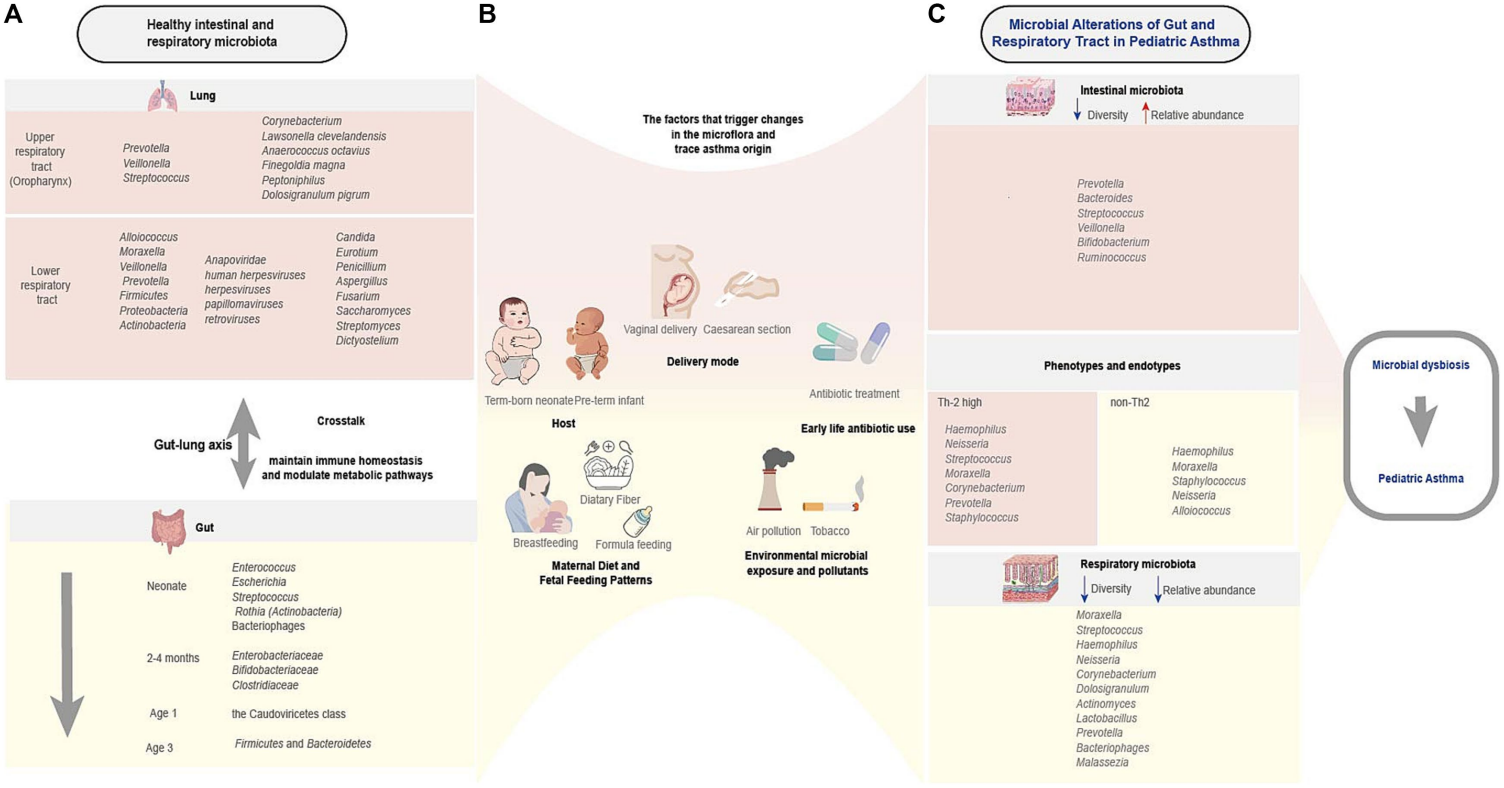
Characteristics of healthy and asthmatic gut-respiratory microbiota and associated influencing factors. This schematic illustrates the interplay between early-life microbial colonization, host-environment interactions, and asthma susceptibility. **(A)** Healthy gut microbiota (e.g., *Firmicutes*, *Bacteroidetes*) and respiratory microbiota (e.g., *Streptococcus*, *Corynebacterium*) undergo dynamic compositional shifts in early life, influencing immune development. **(B)** Multifactorial regulation spans prenatal to postnatal stages: biological factors (delivery mode, maternal diet, infant feeding), ecological exposures (microbial diversity, pollutants), and critical interventions (early antibiotic use), collectively shaping microbiota trajectories. **(C)** Microbial alterations in pediatric asthma and phenotypes and endotypes (Th2-high/non-Th2) correlate with microbial signatures and virome alterations. Bidirectional gut-lung crosstalk (arrows) via metabolic and immune pathways underlies asthma risk. This figure was created by the authors using Adobe illustrator.

Finally, we present Figure 1 to summarize key gut-respiratory microbiota dynamics in pediatric asthma. It delineates healthy colonization patterns, multifactorial influences (e.g., diet, antibiotics), and asthma-associated dysbiosis linked to phenotypes, emphasizing gut-lung crosstalk via immune-metabolic pathways.

## 5 Microbiota-modifying treatments of pediatric asthma

In recent years, groundbreaking advances in microbiome research have made the joint analysis of gut and respiratory microbiomes an important tool for identifying endotypes and patient subgroups in respiratory diseases, paving new avenues for personalized medicine. Based on the theory of the gut-lung axis, current microbiota intervention strategies focus on the regulation of gut microbiota, ranging from traditional probiotics and prebiotics to innovative approaches such as fecal microbiota transplantation (FMT), helminth immunoregulation, phage-targeted therapies, and CRISPR-Cas gene editing technology. These interventions remodel the balance of gut microbiota to regulate pulmonary immune responses, opening up a new path for precise treatment of respiratory diseases while laying a significant foundation for the future development of more targeted therapeutic options.

### 5.1 Probiotics

Probiotics, as foundational interventions, influence asthma progression by modulating gut microbiota balance through the bidirectional gut-lung axis. Representative probiotics such as *Lactobacillus* and *Bifidobacterium* exhibit dual efficacy in preclinical models. For instance, *Lactobacillus* species—particularly *L. rhamnosus*—prevent airway hyperreactivity by reducing eosinophil infiltration (a core pathological feature of asthma) while suppressing type 2 inflammation (Spacova et al., 2020). These effects align with the broader mechanisms by which probiotics (including *Lactobacillus*, *Bifidobacterium*, and *Saccharomyces* spp.) regulate gut-respiratory interactions (Ozdemir, 2010; Sanders et al., 2019). Bifidobacteria regulate early immune system development in infants by metabolizing human milk oligosaccharides (HMOs). Deficiency in HMO-metabolizing capacity within the gut microbiota correlates with heightened Th2- and Th17-driven inflammation (Henrick et al., 2021). Dairy products like yogurt, which enhance probiotic colonization in the gastrointestinal tract, serve as ideal delivery vehicles. Thus, it is recommended that expectant mothers increase their intake of fermented dairy products and prioritize breastfeeding to strengthen probiotic colonization and provide early protection for infant respiratory health.

### 5.2 Nutritional interventions

Nutritional interventions serve as a critical strategy for modulating the development and progression of asthma, encompassing two primary approaches: dietary fiber and nutritional supplements. Dietary fiber intake promotes the production of gut microbiota-derived metabolites, enhancing epithelial barrier function, driving regulatory T cell (Treg) differentiation, and suppressing Th2 polarization and mast cell hyperactivation, thereby synergistically alleviating asthma pathogenesis (Venter et al., 2022). Animal models further demonstrate that dietary fiber supplementation restructures the gut microbiota in asthmatic mice, accompanied by reduced eosinophilic inflammation, decreased Immunoglobulin E (IgE) levels, diminished Th2-associated mediators, and improved pulmonary function (Manni et al., 2021). Notably, maternal dietary fiber intake before and during pregnancy regulates offspring allergic sensitization and airway hyperreactivity through transgenerational microbial programming mechanisms (Thorburn et al., 2015). This vertical microbiota transmission effect provides innovative insights for early-life asthma prevention.

Another category of nutritional interventions—nutritional supplements (defined as bioactive components with health benefits beyond basic nutritional requirements)—exerts antioxidant and anti-inflammatory properties that are closely associated with pulmonary function maintenance (Tuna and Samur, 2025). For instance, prenatal supplementation with vitamin D or polyunsaturated fatty acids (PUFA) has been shown to reduce childhood croup incidence (Brustad et al., 2023), indicating their potential protective role against respiratory disorders. These interventions may indirectly influence asthma trajectories by modulating immune balance and inflammatory responses. Building on existing evidence, future research should further explore the application potential of targeted nutritional interventions, such as specific dietary fiber combinations or precision-based nutritional supplementation protocols, in primary prevention and clinical management of pediatric asthma. This approach aims to establish novel paradigms for asthma prevention and treatment grounded in nutritional regulation.

### 5.3 Fecal microbiota transplantation

In the realm of precision interventions, microbiota transplantation technologies show transformative potential. Fecal microbiota transplantation (FMT) enhances microbial diversity, reshapes gut microbiota composition by increasing *Bacteroidetes* (symbiotic bacteria) and reducing *Proteobacteria* (potential pathogens) (Seekatz et al., 2014; Zheng et al., 2025), and elevates SCFA levels, effectively alleviating airway inflammation in animal models (Lai et al., 2025). A novel whole-intestinal microbial intervention strategy combining healthy donor-derived intestinal fluid transplantation (HIFT) with FMT demonstrates translational potential for pediatric asthma, supported by clinical improvements observed in autism spectrum disorder (Ye et al., 2022). Additionally, emerging virome-targeted strategies reveal that specific temperate bacteriophage taxa in infant guts independently correlate with asthma risk via TLR9-mediated immune interactions (Leal Rodríguez et al., 2024), suggesting that phage therapy to restore temperate virome balance may become an innovative preventive approach.

### 5.4 Emerging microbiota-based therapies

Multiple lines of evidence suggest that helminth infections alter the composition of the intestinal microbiota (Rausch et al., 2018; Pillai et al., 2005). Studies have demonstrated that mice infected with *Heligmosomoides polygyrus bakeri* (a typical parasitic helminth) alter gut microbiota composition by increasing short-chain fatty acids (SCFAs), ultimately alleviating inflammatory responses in dust mite-induced asthma models (Zaiss et al., 2015). Studies have demonstrated that *Heligmosomoides polygyrus bakeri* infection alters gut microbiota composition by selectively promoting the proliferation of SCFA-producing *Clostridiales* while reducing the relative abundance of *Bacteroidales* and *Lactobacillales*, ultimately alleviating inflammatory responses in dust mite-induced asthma models (Zaiss et al., 2015). However, recent research highlights the direct immunomodulatory role of helminths rather than microbiota-dependent mechanisms. Specifically, helminths secrete specific proteins to establish an inhibitory microenvironment within the host, balancing the immune system to attenuate hypersensitivity to allergens (Sun et al., 2019). Therefore, helminth therapy not only provides novel insights into respiratory benefits via gut-lung axis microbiota modulation but also emerges as a promising avenue for asthma prevention and treatment.

As naturally occurring bacterial predators, bacteriophages and their lytic enzymes demonstrate targeted antimicrobial activity with minimal host toxicity, positioning them as viable alternatives against multidrug-resistant pathogens (Guo et al., 2023; MacNair et al., 2024; Wang H. et al., 2024). Research findings on the reduced bacteriophage abundance in asthma patients (Megremis et al., 2023; Choi et al., 2021) suggest that supplementation strategies hold promise for reestablishing microbiome homeostasis in affected individuals. The bacteriophage CRISPR-Cas system, as a defense mechanism against phages and other nucleic acids that invade bacteria and archaea (Dion et al., 2024), demonstrates unique potential through precise editing of bacterial or fungal genomes and targeted elimination of respiratory pathogens. Its delivery system has established a technical paradigm in gut microbiome research, exemplified by the bacteriophage-mediated CRISPR-Cas9 achieving *in situ* knockout of specific genes in *Escherichia coli* (Gencay et al., 2024). For chronic respiratory diseases such as asthma, this system could intervene in disease progression by modifying pathogen virulence genes or regulating host immune responses. Precision strategies combining bacteriophage-CRISPR technologies with patient-specific microbial profiles will advance personalized regulation of respiratory and gut microbiota, pioneering innovative pathways for asthma prevention and treatment.

These interventions collectively establish a microbiota-centric framework for asthma management, offering multi-target immunomodulation with minimal side effects. Future research should focus on optimizing probiotic formulations, advancing clinical translation of microbiota transplantation, elucidating virome regulatory mechanisms, and longitudinally tracking the impact of microbial interventions on disease progression, thereby strengthening the scientific foundation for personalized asthma therapies.

## 6 Conclusion

This study systematically elucidates the critical role of the gut-respiratory microbiome in the pathogenesis of pediatric asthma. It reveals the microbial colonization patterns in normal gut and respiratory tracts and their influencing factors (including host, delivery mode, feeding patterns, antibiotic use, and environmental exposures). The study further analyzes in depth the association mechanisms between specific gut and respiratory microbial dysbiosis and different asthma phenotypes/endotypes. Building on these findings, the study explores innovative microbiome-modulating therapeutic strategies, including probiotic interventions, dietary regulation, and microbiota transplantation. Additionally, it highlights the need for future research to focus on elucidating the precise molecular mechanisms of microbe-immune interactions, providing key insights for developing precision microbiome-based therapies for pediatric asthma.
